# Stage 1-Biomarkers of Kidney Injury in Dogs Undergoing Constant Rate Infusion of Hydroxyethyl Starch 130/0.4

**DOI:** 10.3390/ani11092555

**Published:** 2021-08-31

**Authors:** Barbara Bruno, Roberta Troìa, Francesco Dondi, Cristiana Maurella, Paola Gianella, Ilaria Lippi, Alberto Tarducci, Antonio Borrelli

**Affiliations:** 1 Department of Veterinary Science, University of Turin, Largo P. Braccini 2, 10095 Grugliasco, Torino, Italy; paola.gianella@unito.it (P.G.); alberto.tarducci@unito.it (A.T.); antonio.borrelli@unito.it (A.B.); 2 Department of Veterinary Medical Science, University of Bologna, Via Tolara di Sopra 50, 40064 Ozzano dell’Emilia, Bologna, Italy; roberta.troia2@unibo.it (R.T.); f.dondi@unibo.it (F.D.); 3 Istituto Zooprofilattico Sperimentale del Piemonte, Liguria e Valle D’Aosta, Via Bologna 148, 10154 Torino, Italy; cristiana.maurella@izsto.it; 4 Department of Veterinary Science, University of Pisa, 56122 San Piero a Grado, Italy; ilaria.lippi@unipi.it

**Keywords:** hydroxyethyl starch, renal biomarkers, tubular damage, urinary neutrophil gelatinase-associated lipocalin

## Abstract

**Simple Summary:**

Concern about the use of hydroxyethyl starch (HES) and the development of kidney injury has emerged in human medicine. In veterinary medicine, some retrospective and prospective studies were conducted to investigate the presence of renal damage in dogs using different types and dosages of hydroxyethyl starch. The present study aimed to evaluate the effects of the constant rate infusion of HES 130/0.4 at a dose of 2 mL/kg/h for 24 h on the renal biomarkers of tubular damage and dysfunction. Ten adult dogs with hypoalbuminemia were enrolled, and serum creatinine, fractional excretion of electrolytes, urinary protein to creatinine ratio, urinary albumin to creatinine ratio, qualitative proteinuria, and urinary neutrophil gelatinase-associated lipocalin were measured at the baseline before HES infusion and after 24 and 48 h from the baseline. No significant change in the selected renal biomarkers was observed across time, ruling out the possibility of significant tubular damage after HES 130/0.4 infusion at the dose and rate applied. Further prospective studies are needed to assess the renal safety profile of low-molecular-weight HES administration in more severely affected dogs.

**Abstract:**

In veterinary medicine, investigations relating the effects of hydroxyethyl starch (HES) on renal function report contrasting results. This study aimed to assess the changes in the selected biomarkers of kidney injury in dogs after the administration of HES 130/0.4 as a constant rate infusion (CRI) for 24 h. Ten adult client-owned dogs with hypoalbuminemia (albumin < 2 g/dL) and ongoing fluid losses were included. Enrolled dogs received intravenous fluid therapy with crystalloids and a CRI of HES 130/0.4 at a dose of 2 mL/kg/h for 24 h. Serum creatinine (sCr), fractional excretion (FE) of electrolytes, urinary protein to creatinine ratio (UPC), urinary albumin to creatinine ratio (UAC), SDS-page, and urinary neutrophil gelatinase-associated lipocalin (uNGAL) were measured at the baseline before HES infusion, and after 24 h (T24) and 48 h (T48) from the baseline. No statistically significant difference was found between the baseline value vs. T24 and the baseline vs. T48 for sCr, UAC, UPC, FE of sodium, chloride and calcium, and uNGAL. A significant increase in FEK (*p* = 0.04) was noticed between the baseline and T48. In this study sample of hypoalbuminemic dogs, HES 130/0.4 at the dose and rate of infusion applied did not cause any significant changes in the investigated biomarkers of kidney injury.

## 1. Introduction

Synthetic colloids are a kind of fluid characterized by a large molecular size and are administered for intravascular volume expansion. Hydroxyethyl starches (HESs) are the most used synthetic colloids, and their pharmacological properties (oncotic effect, excretion, and half-life) depend on their mean molecular weight, molar substitution, and C2/C6 ratio. [1] The theoretical benefits of HESs include prolonged intravascular effects, smaller volume requirements, and the reduced risk of tissue edema development compared with crystalloids [1]. However, there is no evidence that the use of any HES solution confers an outcome benefit in critically ill human patients. Moreover, several large-scale human trials have linked HES use with dose-dependent side effects including coagulopathy, nephrotoxicity up to the development of acute kidney injury (AKI), and tissue storage [2,3,4,5,6,7,8]. Despite the claims that the latest HES solution, HES 130/0.4, has a safe profile regarding coagulation and adverse renal effects, the supporting literature is limited [2,3]. There are several suggestions for the mechanisms of HES-induced AKI: a decrease in tubular flow, secondary to the activation of tubuloglomerular feedback, as well as colloid accumulation in the lysosomes of tubular cells that creates an oncotic gradient, leading to the accumulation of intracellular water, cytoplasmic swelling, lysosomal vacuolization, and disruption of cellular integrity [1,4]. The highest HES concentrations have been identified in the proximal renal tubular cells; hence, biomarkers of proximal tubular damage and dysfunction would be ideal to assess the occurrence of HES-induced AKI [4].

In veterinary medicine, three retrospective studies investigated the effects of different HES solutions on renal function and survival in critically ill dogs [9,10,11]. Moreover, three prospective studies assessed renal injury biomarkers during the resuscitation of hypotensive dogs, before and after the bolus of HES 130.04, and other solutions [12,13,14]. Only in the retrospective study from Hayes et al. was HES 250/0.5 administration associated with an increased risk of mortality and AKI occurrence in a dose-dependent manner [9]. In the study of Sigrist et al., the number of days of HES 130/0.4 administration was significantly associated with an increase in AKI grade [11]. On the contrary, in the other studies, therapy with HES 130/0.4 neither resulted in higher serum creatinine (sCr) concentrations, nor in increased urinary biomarkers of AKI, including neutrophil gelatinase-associated lipocalin [10,11,12,13,14]. Few studies with a lack of standardization, small population size, and the use of low sensitivity biomarkers of AKI limit any conclusion concerning the HES safety profile for the kidney in dogs.

The aim of this prospective study was to assess the changes in selected urinary biomarkers of kidney injury in dogs receiving HES 130/0.4 as a constant rate infusion (CRI) for 24 h. We hypothesized that HES 130/0.4 administration in this setting would not be associated with the increase in any assessed biomarker of kidney injury.

## 2. Materials and Methods

### 2.1. Study Design

This study was a prospective investigation performed on client-owned dogs. The protocol was approved by the Bioethics Committee of the University of Turin (Prot. No 26840). The owners of all dogs recruited for participation in the study were informed about the study protocol and signed a consent form.

### 2.2. Animals

Adult dogs referred to the Veterinary Teaching Hospital (Department of Veterinary Science, University of Turin) were enrolled if they had hypoalbuminemia (serum albumin < 2 g/dL) and if they required intravenous fluid therapy for ongoing fluid losses due to their underlying disease. Animals were excluded in the presence of a history of cardiac, pulmonary, renal, and liver failure; positivity to infectious diseases (Snap *Leishmania* Test and Snap 4 Dx (*Dirofilaria immitis* antigen; antibody to *Anaplasma phagocytophilum*; antibody to *Anaplasma platys*; antibody to *Borrelia burgdorferi*; antibody to *Ehrlichia canis*; and antibody to *Ehrlichia ewingi*), IDEXX Laboratories, Westbrook, ME, USA); proteinuria, defined as urine protein to creatinine ratio (UPC) > 0.5 [15]; pyuria, defined as >5 white blood cells per high power field; and the administration of nephrotoxic drugs within 30 days, or drugs known to affect renal solute excretion (e.g., diuretics, hypertonic saline, and isotonic crystalloid) within 48 h before hospitalization.

After the application of a venous catheter in a peripheral vein, fluid therapy with a crystalloid solution (Ringer’s Lactate, Baxter, Roma, Italy) was started while considering maintenance, dehydration, and ongoing losses. To support the colloid osmotic pressure, HES 130/0.4 (Voluven, Fresenius Kabi Italia, Isola della Scala, Italy) was added as a CRI of 2 mL/kg/h (48 mL/kg/day) [16,17]. Dogs were included in the study if the colloid CRI was continued for 24 h, and neither change in the treatment plan nor the administration of nephrotoxic agents occurred during the study period.

### 2.3. Sample Collection, Clinicopathological Data, and Laboratory Methods

Before the administration of HES CRI (T0), the following analyses were performed: a complete blood count (ADVIA 120 Hematology, Siemens Healthcare Diagnostics) with blood smear evaluation (CBC); serum chemistry profile (including the measurement of sCr, urea, albumin, glucose, alkaline phosphatase, aspartate aminotransferase, alanine aminotransferase, and γ-glutamyl transpeptidase) (ILAB 300 plus, Clinical Chemistry System, Instrumentation Laboratories S.p.A., Milano, Italy); venous blood gas analysis (including lactate and electrolyte concentration) (ABL 800 Flex; A. DE MORI S.p.A., Milano, Italy); packed cell volume (PCV) and total proteins (TP); urinalysis including urine specific gravity (USG) (Reichert VET 360, Reichert technologies analytical instrument, Buffalo, NY, USA); dipstick examination (Multistix 10 SG Reagent Strips, Siemens Healthcare Diagnostics, Milano, Italy); microscopic evaluation of the urine sediment and urine chemistry including urine creatinine (uCr), the urine protein to uCr ratio (UPC), urinary electrolytes, urea and glucose, urinary neutrophil gelatinase-associated lipocalin (uNGAL) (Dog NGAL ELISA Kit, BIOPORTO Diagnostics, Needham, MA, USA); and urine sodium-dodecyl-sulfate polyacrylamide gel electrophoresis (SDS-page) evaluation (NuPage, Thermo Fisher Scientific, Waltham, MA, USA).

Analyses for PCV, TP, venous blood gas, albumin, sCr, and complete urinalysis were repeated after 24 h (T24). In 8 dogs, the HES CRI was discontinued at T24, and the previous evaluations were also conducted after 48 h from T0 (T48).

Urine samples were collected by spontaneous voiding or cystocentesis, and an aliquot of fresh urine sample was submitted to the laboratory for urinalysis, including urine specific gravity and dipstick evaluation. Urine sediment obtained by centrifugation (1000× *g* 10 min) was examined within 1 h after collection or within 4 h, keeping the sample at 4 °C. Glucosuria was assessed as the urine glucose to uCr ratio. The fractional excretion (FE) of urine analytes, including sodium (FENa), potassium (FEK), chloride (FECl), calcium (FECa), and urea, was calculated according to the following equation: FEX = (uX sCr/uCr sX) × 100 (based on spot urine sample), where uX and sX are the concentrations of a specific analyte in urine and serum, respectively [18,19].

An SDS-page analysis was performed as previously reported [18], and the concentration of urine albumin (uAlb) and the uAlb to uCr ratio (UAC) were evaluated.

Urine NGAL concentrations were determined on aliquots of the urine supernatant stored at −80 °C for up to 6 months until assayed, as previously reported [19]. The storage time was established according to previous studies [20]. NGAL was measured using a commercial ELISA sandwich assay according to the manufacturer’s instructions (Dog NGAL ELISA Kit, BIOPORTO Diagnostics, Needham, MA, USA). The results are expressed as an absolute uNGAL concentration (pg/mL) and as an uNGAL to uCr ratio (uNGALC, pg/mg).

### 2.4. Statistical Analysis

The sample size for repeated measures was determined according to the power at 80%, confidence level at 95%, the expected difference between control group (T0) and treated group (T24) equal to a 150% increase of uNGALC, and the difference between standard deviations equal to 1.

The sample size calculation was based on evidence that an 800-fold increase in uNGALC is associated with the development of AKI in dogs [19]. Thus, the inclusion of at least 8 dogs was considered necessary to identify an increase of 1,5-fold of uNGALC from T0 to T1.

Prior to the test selection, data were assessed for normality graphically and with the Shapiro-Wilk test. Descriptive statistics were evaluated as appropriate, and data were presented as the median and range (min-max). Paired data (T0 vs. T24; T24 vs. T48; an T0 vs. T48) were compared using a Wilcoxon matched-pairs signed-rank test if not normally distributed, otherwise a t-test was used. Results were considered significant if *p* < 0.05. 

Statistical analyses were performed using a Stata 15.1 (StataCorp 16.1, 4905 Lakeway Drive Special Edition College Station, Texas, TX, USA).

## 3. Results

### Study Population

A total of 10 dogs were included and completed the study protocol. The study population was composed of two intact males, six intact females and two spayed females, with a median age of 7 years (min 1–max 12). The median body weight was 26.7 kg (min 5–max 39) and the breeds included mixed breeds (*n* = 3), Jack Russel Terriers (*n* = 2), and one each of Rottweiler, Labrador Retriever, Dachshund, Hound, and English Bulldog. The underlying pathologies affecting the dogs were protein losing enteropathy (7/10 dogs), septic peritonitis (1/10 dogs), chylothorax (1/10 dogs), and hypoadrenocorticism (1/10 dogs).

No statistically significant difference was found between the baseline value vs. T24, T24 vs. T48, and T0 vs. T48 for sCr, UAC, UPC, uCr/sCr, FENa, FECl, FECa, uNGAL, and uNGALC (Table 1).

A significant increase in TP (*p* = 0.04) and the urine glucose to uCr ratio was observed between T0 vs. T24. The serum albumin concentration increased significantly between T24 vs. T48 (*p* = 0.03) and between T0 vs. T48 (*p* = 0.01). Finally, a significant increase in serum glucose (*p* = 0.01) and FEK (*p* = 0.04) was observed between T0 vs. T48. All results are reported in Table 1.

## 4. Discussion

The present study did not show any significant change in the selected biomarkers of kidney injury in dogs receiving a CRI of 2 mL/kg/h of HES 130/0.4 for 24 h. Hence, in our study population of hypoalbuminemic dogs, the administration of low-molecular-weight HES 130/0.4 at the dose and rate of infusion applied was not associated with signs of tubular damage or dysfunction, at least recognizable with the investigated biomarkers in the time frame chosen for monitoring.

In critically ill humans, HES exposure has been linked to an increased incidence of AKI and the need for renal replacement therapy, particularly in patients affected by sepsis or burns, whereas studies conducted in patients undergoing surgery have reported contrasting results [7,21,22,23,24,25,26]. Datzmann et al. assessed some functional renal parameters and structural biomarkers (including NGAL) in people undergoing coronary bypass grafting without showing differences in mortality, acute kidney injury, the need for renal replacement therapy, or evidence of a mechanism for tubular injury [25]. Momeni et al. and Kashy et al. detected a correlation between the dose of colloids used and the incidence of AKI after surgery. Unfortunately, such results are not comparable due to heterogeneous populations, the type of colloid used (old vs. new generation), the dose administered, and the definition of AKI applied [23,26].

The suggested pathogenesis of HES-induced AKI is multifactorial and not fully understood. HES uptake and accumulation in the renal tissue cause renal storage lesions, deemed osmotic nephrosis lesions. They are characterized by interstitial macrophage infiltration, intracellular water accumulation, cellular swelling, disruption, and death, and mainly involve proximal tubular cells at histological examination [26]. It was reported that the number of colloid molecules and cumulative doses are major factors responsible for proximal tubular cell injury, especially where older HESs with a high molecular are used (10% HES 200/0.5) [26].

In veterinary medicine, most of the information about HES doses, the modality of administration, and the side effects has been derived from the human literature, except for their use as a CRI. The latter has been reported in animals only and originated from the recommended daily maximum doses of 20–30 mL/kg/day suggested for older generations of HESs [16,17]. However, extrapolating guidelines from human studies to regulate the use of HESs in dogs may be inappropriate due to inherent metabolic differences between the two species; indeed, dogs have a greater α amylase activity, potentially allowing for a greater capacity to degrade HESs. The consequence could be a decrease in colloid accumulation in the renal tissue and a reduction in the associated injury [2]. Nonetheless, the studies investigating HES-induced AKI in dogs are often retrospective in nature and difficult to compare due to the few numbers of cases included and the different biomarkers used to assess AKI [9,10,11,12,13,14]. In the retrospective study by Hayes et al., the administration of older HES with a high molecular weight (HES 250/0.5) was associated with adverse effects like death or AKI, with higher HES doses being associated with an increased risk [9]. Yozova et al. and Sigrist et al. observed no significant short-term increase in sCr concentrations following HES 130/0.4 administration in a population of canine intensive care patients [10,11]. Nonetheless, in the latter study, the duration of HES administration was significantly associated with an increase in AKI grade within 10 days [11]. Three recent prospective canine studies evaluated several biomarkers of AKI, including plasma and urine NGAL, during the infusion of different solutions for volume replacement [12,13,14]. Hypotensive dogs resuscitated with bolus injections of HES 130.04 did not show a significant increase in NGAL concentrations [12,13,14]. In particular, Boyd et al. documented only nonsignificant marginal increases in numerous biomarkers of renal tubular damage other than NGAL. In addition, no greater histological tubular injury was documented in dogs receiving HES compared to other resuscitation fluids, including natural and synthetic colloids [13]. Another recent prospective randomized controlled trial did not show differences in the urine NGAL, cystatin C, or kidney injury molecule-1 in dogs with shock before and after a fluid bolus of Hartmann’s solution or HES 130.04 [14]. Thus, an increased likelihood of AKI was reported in particular when older hyperoncotic HES solutions (HES 250/0.5) were used in dogs with sepsis, trauma, or surgery [9]. However, conclusions regarding HES safety in dogs cannot be made since most of the cited studies were underpowered to document small variations in sCr or kidney injury biomarkers in study populations where the overall likelihoods of AKI and sepsis were low.

Although the current study does not overcome such limitations, the prospective design, the unstudied method of HES infusion (CRI), and the panel of biomarkers used to assess tubular damage and dysfunction represent potential strengths.

The NGAL is a protein expressed by neutrophils and many epithelial cells, including the renal tubular ones [27]. The expression of uNGAL is rapidly induced in nephrons in response to renal epithelial injury and inflammation, and concentration seems to be correlated with glomerular filtration rate [28,29]. Its increase in urine is reported to anticipate the rise in sCr concentration, and after the validation of a canine-specific NGAL ELISA kit, several studies investigating uNGAL sensitivity, specificity, and kinetics to diagnose and prognosticate AKI in dogs have been published [19,28]. According to the results of a recent study evaluating uNGALC between healthy dogs and dogs with AKI, this variable can be considered a sensitive marker of AKI, showing up to an 800-fold increase in the case of intrinsic AKI [19].

The lack of increase in uNGALC values after HES infusion in the enrolled dogs could rule out the development of significant glomerular and tubular injury in our setting, both at T24 and T48, in line with previous studies investigating low-molecular-weight HES in different scenarios [10,11,12,13,14].

Similarly, the FE of electrolytes recently has shown the potential to characterize and prognosticate AKI [18,19]. It should be pointed out that the FE of electrolytes acts both as a marker of tubular damage and function, but its sensitivity and specificity to detect renal injury is currently unknown. Although fluid therapy has historically been reported to affect urinary electrolytes measurements and confound their interpretation, no significant increases in FENa, FEK, FECl, and FECa were documented in our patients at T24, after HES and Ringer’s Lactate infusion. Only a slight but significant increase in FEK was documented between T0 and T48. The reported value for normal FEK in dogs is <20%, and a marked increase was observed in a recent study evaluating dogs affected by AKI (>100%) [30]. In our opinion, it is difficult to relate the increase in FEK noticed in our study to the development of tubular injury. The median values of FEK measured in our study population ranged from 6% to 10% and could still be considered normal. In addition, the distal tubule is primarily implicated in K excretion and less susceptible to damage during AKI compared with the proximal tubule, and none of the other biomarkers evaluated indicated tubular damage. Similarly, the slight but significant increase in the urine glucose to uCr ratio observed between T0 vs. T24 was not considered clinically relevant because it was within the reference range.

SDS-PAGE represents a reliable and specific technique to investigate tubular proteinuria eventually related to tubular damage. The lack of any relevant increase in quantitative and qualitative proteinuria in the pre- and post-HES infusion measurements further corroborates that HES 130.04 infusion does not cause an increase in tubular proteinuria in our study conditions.

There are some limitations to consider when interpreting our results. The study population was mainly composed of noncritical dogs, as ICU hospitalization was not a criterion required for enrollment. Similarly, the number of septic patients was extremely low (1/10). Hence, the inclusion of a subgroup of more severely affected patients could have led to different results in terms of an increase in renal biomarkers. Moreover, this was designed as a single-arm trial, as each enrolled case served as its own control and no different control group was available. Therefore, despite the collection of additional safety data for HES 130/0.4 in our setting, these should be confirmed in large-scale randomized controlled trials. It might also be possible that HES infusion in our study did not cause detectable kidney injury due to the limited time of infusion chosen. Long-term monitoring may be as important as short-term monitoring, since long-term effects caused by HES accumulation in renal tubular cells are expected after 20 days from HES exposure in people, and renal function monitoring up to 90 days after HES administration is advocated [4]. The effects of larger HES doses or longer infusion times was not addressed, as that was beyond the scope of the study. Finally, no histopathological data were available to assess the presence and severity of tubular damage.

## 5. Conclusions

In conclusion, in this study sample of hypoalbuminemic dogs, no significant changes in selected biomarkers of tubular damage and dysfunction were observed after HES130/0.4 infusion at the dose and rate applied. Further prospective studies are needed to assess the renal safety profile of low molecular weight HES administration considering different dose regimens, long-term monitoring, and including a subgroup of more critically ill dogs.

## Figures and Tables

**Table 1:** Descriptive statistics reported as the median and range (minimum–maximum value) and comparison for clinicopathological variables among samples collected at T0 (pre-HES infusion), at T24 (after 24 h of HES continuous rate infusion), and at T48 (after 48 h from baseline and after HES CRI interruption).

Variable	T0 (N = 10)	T24 (N = 10)	T48 (N = 8)	Institutional Reference Values	*p* *-Value	*p* ^#^-Value	*p*^§^-Value
**Glucose** (mmol/L)	5.6 (4–7.9)	5.2 (4.4–7.2)	5.7 (4.8–7.2)	4.1–7.4	1	1	0.008
**Creatinine** (µmol/L)	59.2 (46–73.4)	45.1 (33.6–75.1)	50.4 (34.5–81.3)	44.2–132.6	0.18	0.29	0.73
**Chloride** (mmol/L)	119 (107–124)	120 (107–124)	122 (113–124)	109–120	0.45	1	1
**Sodium** (mmol/L)	143 (123–148)	143 (130–150)	141 (133–150)	140–150	0.29	0.63	0.69
**Potassium** (mmol/L)	4 (3.4–5.7)	3.85 (3.5–5.5)	4.05 (3.5–5)	3.9–4.9	1	1	1
**Ionized Calcium** (mmol/L)	1.2 (1.1–1.3)	1.3 (1.1–1.4)	1.3 (1.1–1.3)	1.25–1.5	0.11	0.63	0.13
**UPC**	0.1 (0.02–0.29)	0.07 (0.04–1.36)	0.13 (0.02–0.2)	≤0.5	1	1	0.7
**UAC**	0.01 (0.0004–0.07)	0.013 (0.001–0.06)	0.01 (0.001–0.07)	0.00-0.03	0.34	0.73	0.73
**uCr** (µmol/L)	12,959 (3960–49,707)	11,386 (2997–64,665)	136.9 (31.4–625.6)	3894–49,769	0.75	0.3	0.73
**uCr**/**sCr**	146.4 (44.8–562.3)	128.8 (33.86–731.5)	136.86 (31.4–625.56)	37–547	0.75	0.14	0.73
**uNGAL** (pg/mL)	972.8 (154.5–38,991.8)	1271.35 (62.1–62,540.9)	1656.45 (201.2–140,572.8)	0–2600	1	1	0.73
**uNGALC** (pg/mg)	0.08 (0.026–3.96)	0.27 (0.02–12.76)	0.16 (0.05–4.49)	0–1200	1	0.73	0.73
**uGLUCr**	0.10 (0.06–0.26)	0.14 (0.084–0.37)	0.11 (0.05–0.3)	0–0.6	0.01	0.29	1
**FENa** (%)	0.21 (0.018–2.98)	0.79 (0.016–3.67)	0.61 (0.04–2.84)	0–0.69	1	1	0.7
**FECl** (%)	0.26 (0.05–3.55)	1,08 (0.03–4.23)	0.59 (0.06–3.14)	0–1.09	1	0.45	0.13
**FEK** (%)	6.86 (2.96–21.4)	6.6 (0.97–12.23)	10.53 (3.09–16.33)	2.3–23.8	0.75	0.28	0.04
**FECa** (%)	1 (0.32–8.47)	1.14 (0.16–6.9)	0.59 (0.19–2.16)	0–0.33	0.75	1	1

FECa, fractional excretion of calcium; FECl, fractional excretion of chloride; FEK, fractional excretion of potassium; FENa, fractional excretion of sodium; PCV, packed cell volume; UAC, urine albumin to creatinine ratio; uCr, urine creatinine; uCr/sCr, urine creatinine to serum creatinine ratio; uGLUCr, urine glucose to creatinine ratio; uNGAL, urinary neutrophil gelatinase-associated lipocalin; uNGALC, uNGAL to uCr ratio; UPC, urine protein to creatinine ratio; USG, urinary specific gravity; * difference between T0 and T24; ^#^ difference between T24 and T48; ^§^ difference between T0 andT48.; *p* < 0.05 indicate statistically significant difference.

## Data Availability

All data analyzed during this study are included in this published article.
